# Combination of Paper and Electronic Trail Making Tests for Automatic Analysis of Cognitive Impairment: Development and Validation Study

**DOI:** 10.2196/42637

**Published:** 2023-06-09

**Authors:** Wei Zhang, Xiaoran Zheng, Zeshen Tang, Haoran Wang, Renren Li, Zengmai Xie, Jiaxin Yan, Xiaochen Zhang, Qing Yu, Fei Wang, Yunxia Li

**Affiliations:** 1 Department of Neurology Tongji Hospital, School of Medicine Tongji University Shanghai China; 2 Department of Computer Science and Technolgy College of Electronic and Information Engineering Tongji University Shanghai China; 3 Department of Neurosurgery Tongji Hospital, School of Medicine Tongji University Shanghai China

**Keywords:** cognition impairment, Trail Making Test, vector quantization, screening, mixed mode, paper and electronic devices

## Abstract

**Background:**

Computer-aided detection, used in the screening and diagnosing of cognitive impairment, provides an objective, valid, and convenient assessment. Particularly, digital sensor technology is a promising detection method.

**Objective:**

This study aimed to develop and validate a novel Trail Making Test (TMT) using a combination of paper and electronic devices.

**Methods:**

This study included community-dwelling older adult individuals (n=297), who were classified into (1) cognitively healthy controls (HC; n=100 participants), (2) participants diagnosed with mild cognitive impairment (MCI; n=98 participants), and (3) participants with Alzheimer disease (AD; n=99 participants). An electromagnetic tablet was used to record each participant’s hand-drawn stroke. A sheet of A4 paper was placed on top of the tablet to maintain the traditional interaction style for participants who were not familiar or comfortable with electronic devices (such as touchscreens). In this way, all participants were instructed to perform the TMT-square and circle. Furthermore, we developed an efficient and interpretable cognitive impairment–screening model to automatically analyze cognitive impairment levels that were dependent on demographic characteristics and time-, pressure-, jerk-, and template-related features. Among these features, novel template-based features were based on a vector quantization algorithm. First, the model identified a candidate trajectory as the standard answer (template) from the HC group. The distance between the recorded trajectories and reference was computed as an important evaluation index. To verify the effectiveness of our method, we compared the performance of a well-trained machine learning model using the extracted evaluation index with conventional demographic characteristics and time-related features. The well-trained model was validated using follow-up data (HC group: n=38; MCI group: n=32; and AD group: n=22).

**Results:**

We compared 5 candidate machine learning methods and selected random forest as the ideal model with the best performance (accuracy: 0.726 for HC vs MCI, 0.929 for HC vs AD, and 0.815 for AD vs MCI). Meanwhile, the well-trained classifier achieved better performance than the conventional assessment method, with high stability and accuracy of the follow-up data.

**Conclusions:**

The study demonstrated that a model combining both paper and electronic TMTs increases the accuracy of evaluating participants’ cognitive impairment compared to conventional paper-based feature assessment.

## Introduction

By 2030, the number of people with dementia is expected to reach 78 million worldwide. However, Alzheimer's Disease International estimates that 75% to 90% of people with cognitive impairment may not be diagnosed, especially in some low- and middle-income countries [1]. The insufficient number of trained clinicians and lack of attention to dementia remain major barriers to diagnosis. In addition, the partial lockdown and even shutdown of most countries to contain the spread of COVID-19 during 2020 to 2021 have worsened the situation [2]. As the world’s older population continues to grow, the individual and societal burdens of dementia and age-associated diseases will increase substantially in the coming years. Digital assessment tools can increase the efficiency and reduce the demand for trained clinicians [3]. Further, they offer more reliable and reproducible results by standardizing data collection and processing procedures [4]. Several short screening scales specially designed to detect cognitive decline are useful for increasing the diagnosis rate and fostering appropriate and timely support for individuals with dementia [5]. Electronic tests based on these short screening scales have been designed as fast and useful screening tools for rapid testing or self-testing of cognitive impairment, which can provide support for the early identification of at-risk older individuals at home.

The Trail Making Test (TMT) is a neuropsychological test that evaluates psychomotor speed by connecting numbers as quickly and accurately as possible, as well as the ability of set shifting (also called task shifting), which involves the ability to alter a response in the face of change [6,7]. The TMT consists of 2 parts: TMT-A [8], which require participants to connect numbers in ascending order, and TMT-B [9], which introduces an additional task associated with alternating sequences. Traditional assessment methods are based on paper and pencil and obtain the test score via the static information of the drawing outcome and subjective physician judgment. However, the TMT does not solely reflect frontal execution in cognitive impairment [9,10]. Recent findings suggest that impairments in executive function and working memory may also be critical indicators of mild cognitive impairment (MCI). Subtle deficits in these cognitive functions might occur years before the clinical diagnosis of Alzheimer disease (AD) [11].

In the past decades, the TMT has proven to be sensitive to cognitive changes and has been adapted in many countries, including the United States, China, France, and Brazil. However, TMT error analysis does not appear to provide additional diagnostic utility for subjective cognitive decline, MCI, or AD [12]. Computerized technology has recently gained increasing attention and has been used to support both quantitative assessments of cognitive decline and continuous patient monitoring [13]. A wide variety of computerized neurocognitive tasks have been explored using iPads (Apple Inc), laptops, and tablets with touch-sensitive screens or external touchpads [4,14]. Furthermore, extensive research has investigated novel approaches using smartphones for cognitive assessment, given the increased use of mobile technology by older adults and the reduced financial burden it entails. However, opponents of digital assessment argue that even a slight format change in paper-based assessment may result in significant differences in the measured performance of patients to perceive or respond to computer-generated and paper stimuli [15]. In addition, familiarity with computer interfaces (eg, keyboard, mouse, and touchscreen) becomes an independent variable that is unrelated to the experimental design.

However, with the development of artificial intelligence technology, many shortcomings of traditional cognition assessments can be overcome [13,15]. Several studies have found that a comprehensive assessment of cognition can reflect a patient’s real status [16]. Although many studies have focused on developing models based on the Clock Drawing Test [17,18] and Rey Complex Figure Test [19,20], there is a relative scarcity of studies based on the TMT. However, the TMT is a promising test to represent the trajectory of cognitive decline [21]. This study aimed to explore the mixing of paper and electronic TMTs for the assessment of cognitive impairment that attempts to maximize similarities to traditional tests. We also developed an appropriate machine learning method to capture task-relevant features from recorded hand-drawn trajectories, evaluated the validity and effectiveness of the proposed framework for the detection of MCI and dementia, and compared its performance with that of the conventional assessment method.

## Methods

### Participants

All participants were recruited and evaluated at the Tongji Hospital, affiliated with Tongji University, Shanghai, China. Data collection took place between January 2018 and October 2021. Following the comprehensive neuropsychological assessment tests based on 2011 National Institute on Aging-Alzheimer’s Association guidelines [22], brain magnetic resonance imaging or computed tomography scan, and serum blood tests, our team, comprising board-certified psychiatrists and neurologists, categorized participants into 3 groups: healthy controls (HC), participants diagnosed with MCI, and participants diagnosed with AD. The exclusion criteria were as follows: (1) any lifetime history of stroke, head injury, substance abuse, or major or medical psychiatric disorders; (2) large intracranial vessel stenosis >50%; and (3) being unable to cooperate with neuropsychological tests. We conducted a 1-year follow-up to assess their cognitive function, including executive function (TMT), and re-evaluated their cognitive diagnosis and executive function.

### Ethics Approval

Ethics approval (#2021-081) was granted by the institutional review board of the Ethics Committee of Shanghai Tongji Hospital in China and complied with the principles of the Declaration of Helsinki. All participants were informed about the study and the confidentiality of their data and signed a consent form before participating in the study at baseline and follow-up. All data used in this analysis were deidentified. There was no compensation for participating in this study.

### Protocol

**Figure 1:**
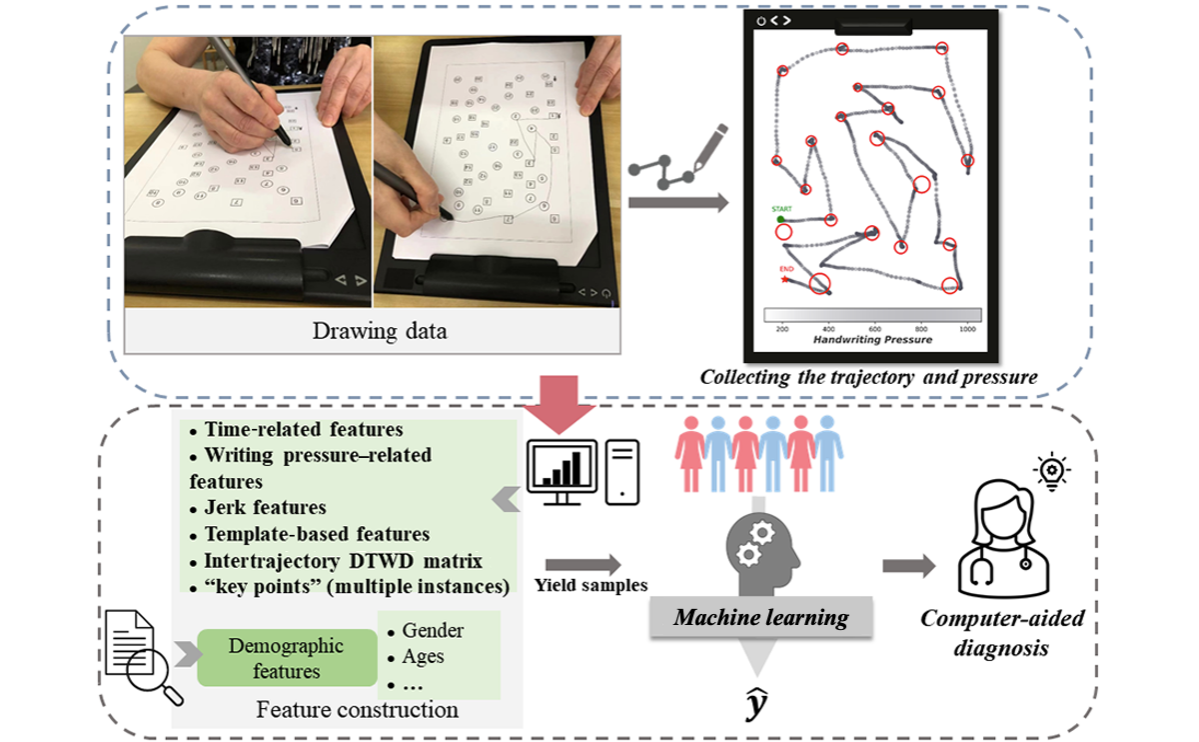
Study flowchart. The combination of paper and electronic Trail Making Tests (TMTs): A4 paper placed on an electromagnetic tablet. Participants performed the TMT using a digital pen, and the electromagnetic tablet collected the trajectory and pressure. Subsequently, the automated analyzing program extracted features highly related to brain dysfunctions to facilitate diagnosis.

The combination of paper and electronic TMTs retains the same psychometric properties as the standard TMT but provides more abundant information for quantitative assessment, such as wrist velocity and pressure level. In this study, participants were given a printout of the TMT on A4 paper and were instructed to draw lines to connect the circles or squares in ascending order. The A4 paper was placed on an electromagnetic tablet (PH-1820-A; PendoTech). This combination approach not only records abundant information but also provides a better interactive experience for older adults who are not familiar or comfortable with electronic presentations. The trajectory points collected by the digital tablet consisted of 4 variables, including Cartesian coordinates (x and y), pressure, and time stamps. The Cartesian coordinates represented the position of the trajectory points on a 2D plane, where x ranged from 0 to 21,000 and y ranged from 0 to 29,700 because of the limited tablet size (210 × 297 mm). The pressure variable indicates the strength of the drawing, and the time stamp is the sampling time of the point. When the pen is lifted in the air, the pressure becomes zero, which is highly beneficial for calculating the preparation time or thinking time. A flowchart of the study is shown in Figure 1.

The TMT was initially developed by Zhao et al [23] in 2013 with the aim of eliminating reliance on the Latin alphabet. In this study, the TMT (also called the Shape Trial Test) assessment had a standardized format and was formulated and administered by well-trained neuropsychologists. In Part A, the participants were instructed to draw a line between circles or squares as rapidly as possible, joining consecutive numbers. Part B displayed all the numbers twice, except for the number 1 (encircled by a square), with each corresponding number encompassed in both circles and squares. Hence, Part B was more demanding than Part A for visual perceptual processing ability because of greater visual interference and longer path length [19]. Parts A and B had 2 blocks: in the first block, the numbers ranged from 1 to 8, and in the second block, the numbers ranged from 1 to 25. In the first block, the participants were allowed to attempt the test without restraint. The short warm-up blocks introduced participants to an understanding of the TMT-square and circle. However, in the second block, several rules were introduced to regulate the participants’ behavior. Similar to the paper-based TMT, if a connection error occurred, the examiner pointed it out and allowed the participant to correct it. When the participant was confused about the next target to connect for more than half a minute, the examiner prompted the participant. In addition, to record complete trajectories, participants were cautioned whenever they lifted the pen from the paper. Finally, the number of connection errors, prompts, and pen-up warnings was recorded.

### Data Analysis

#### Overview

Participants underwent the TMT assessment, which was conducted by multiple interns blinded to the diagnosis. We used an automated drawing analysis procedure to extract features that were highly related to brain dysfunction. The analysis involved 2 parts. The first part was the cross-sectional data, which included HC (n=100), participants diagnosed with MCI (n=98), and those diagnosed with AD (n=99). The second part was the follow-up data, which included 38 HC, 32 participants with MCI, and 22 participants with AD. These key features included (1) time-related features, (2) writing pressure–related features, (3) jerk features, and (4) template-based features.

#### Time-Related Features

Time-related features are commonly used in the TMT. Extensive research has confirmed that several factors influence the time required to complete a trial, such as visual search and scanning ability, motor planning and execution, and error correction. Hence, we recorded the completion time of each block as well as the time of motor preparation and execution. Meanwhile, TMT-A and TMT-B provide different measures of cognitive flexibility, so the difference in the completion time between TMT-A and TMT-B is significantly correlated with cognitive impairment level. Here, the difference and ratio of completion times between the TMT-A and TMT-B were calculated as the analysis features.

#### Writing Pressure-Related Features

Writing pressure can reflect the hand-muscle strength and handgrip strength of patients. Particularly, in patients with paralysis, the pressure is low. In this study, we calculated the mean, minimum, and maximum writing pressures for each block.

#### Jerk Feature

An important characteristic of human movement is the minimum jerk, where jerk indicates the time derivative of acceleration. Hence, we leveraged jerk as an empirical measure of smoothness to evaluate patient-hand vibrations likely caused by ataxia.

#### Template-Based Features

This study assumed that every trajectory in the HC group contained components similar to the standard answer. Hence, we can select a trajectory that is highly similar to all the others within the HC group as the optimal template (or reference). Specifically, a template-selection rule was used to select the structure with the highest sequence similarity. First, we randomly chose 10 trajectories from the control group of the training set to form a subset and then calculated the intertrajectory dynamic time warping distance (DTWD) within the subset [24,25].

The trajectory was selected as the reference if the sum of the intertrajectory DTWD with the other trajectories was the smallest. Finally, the reference trajectory was Gaussian smoothed with a sigma value of 0.1.

**Figure 2:**
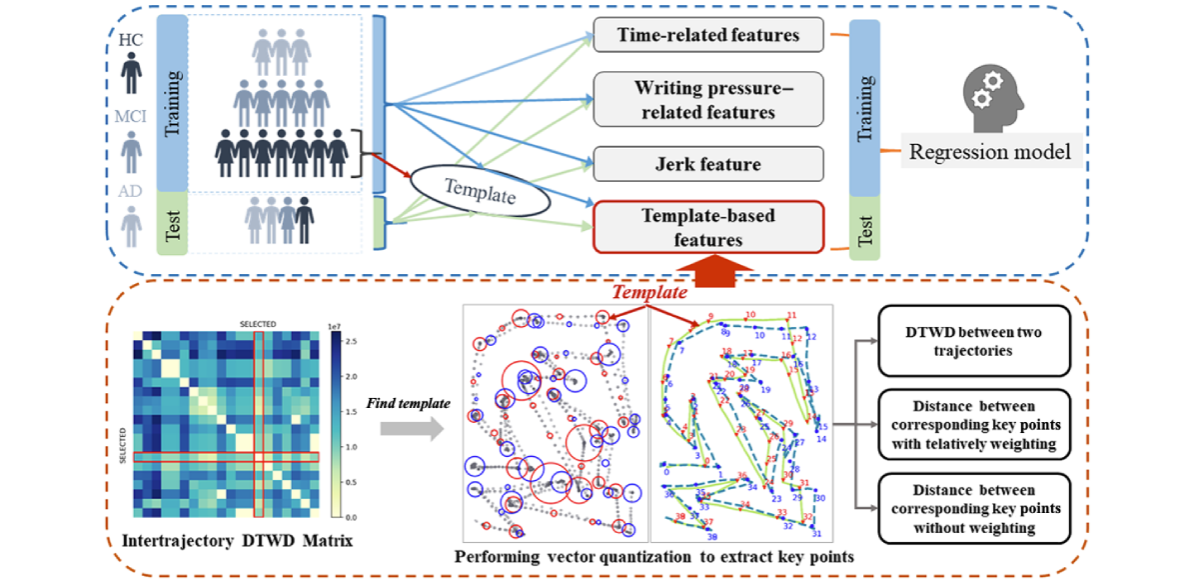
Workflow for feature extraction. A 5-fold cross-validation was introduced, where each fold served once as the test set to validate the performance of the model while the remaining 4 folds were used to train the model. In other words, the validation was run 5 times, each run with a different fold as the test set. The reported metrics (area under the curve, accuracy, etc) are the mean values of these 5 runs. Meanwhile, the template-based feature extractor required special treatments. First, we calculated intertrajectory DTWD within the HC group and selected the trajectory that had minimum average DTWD as the template. Second, we performed clustering and vector quantization to extract key points of the template and all other trajectories. Finally, each trajectory was compared with the template to obtain template-based features. AD: Alzheimer disease; DTWD: dynamic time warping distance; HC: health controls; MCI: mild cognitive impairment.

Next, we performed clustering and vector quantization (VQ) on the reference and all trajectories of both the training and testing sets to extract the key points. The codebook size was set to 40. Clusters of points were found, based solely on the spatial distribution, regardless of the time factor. Therefore, a few points distant from the clustering center in time may be assigned to the cluster, which in turn causes a deviation in the clustering center. Here, we restructured the point set within each cluster by removing points earlier or later than the mean time of the last or next cluster, respectively. Subsequently, we recalculated the arithmetic mean of the point set as a new clustering center (also called key points). Eventually, we extracted template-based features based on these key points, which included the DTWD and Euclidean distance with or without relative weighting between the corresponding key points between the reference and all trajectories. Relative weights were used to quantify the number of points in the corresponding clusters relative to the trajectory length. The following Python (version 3.7; Python Software Foundation) libraries were used to extract template-based features: *FastDTW* [24,25] (version 0.3.4, mainly devoted to DTWD computing) and *scikit-learn* (version 0.24.2; mainly *scipy.cluster.vq.kmeans2* for VQ). The workflow of the proposed feature extraction method is illustrated in Figure 2.

## Results

### Demographic Characteristics

The entire cohort (N=300) was stratified and randomly sampled into a subset for model development and validation, with 100 participants in each group. Three participants with incomplete data were excluded. The demographic characteristics of the participants enrolled in the study are shown in Table 1. We used chi-square tests and one-way ANOVA to assess the independence of categorical variables between each pair of groups. A highly significant association was observed between the HC and AD groups in terms of age (*P*<.001) and educational level (*P*<.001) and between the HC and MCI groups for age (*P*=.24). In addition, there were no significant between-group differences for sex (*P*=.06).

**Table 1:** Demographic characteristics of participants in this study.

Characteristic	HC^a^ group (n=100)	MCI^b^ group (n=98)	AD^c^ group (n=99)	*P* value			
				HC vs MCI	HC vs AD	MCI vs AD	Between-group difference
Sex, female, n (%)	47 (47)	56 (57)	36 (36)	.19	.27	.43	.06
Age (years), mean (SD)	69.83 (6.92)	72.47 (7.45)	72.44 (8.67)	.24	<.001	.02	<.001
Education (years), mean (SD)	12.46 (3.53)	10.00 (4.01)	8.32 (5.17)	<.001	<.001	.09	<.001

^a^HC: healthy controls.

^b^MCI: mild cognitive impairment.

^c^AD: Alzheimer disease.

### Evaluation Outcomes

To investigate the associations between each feature and cognitive decline associated with cognitive impairment, a one-way ANOVA was used to explore whether differences existed between the groups. As shown in Table 2, time-related and writing pressure–related features were significantly associated with cognitive ability (*P*<.001), whereas a lower significance for the jerk feature indicated a weak discriminating ability (TMT-A-1, HC vs AD, *P*=.57). Meanwhile, for TMT-B-2 in particular, the higher significance indicated that the TMT-B-2 had a more powerful ability to identify cognitive impairment in patients (*P*<.001).

**Table 2:** Analysis of the association between each feature and cognitive impairment using one-way ANOVA.

Features			HC^a^ group (n=100)		MCI^b^ group (n=98)		AD^c^ group (n=99)		*P* value						
									HC vs MCI		HC vs AD			MCI vs AD	
**Completion time**															
	TMT^d^-A-1	13.135		19.428		32.320		<.001			<.001		<.001		
	TMT-A-2	53.650		72.996		89.671		<.001			<.001		.03		
	TMT-B-1	27.348		47.991		39.920		<.001			.002		.12		
	TMT-B-2	138.307		164.322		72.415		.001			<.001		<.001		
	Time difference between TMT-A-2 and TMT-B-2	–88.250		–91.330		10.469		.71			<.001		<.001		
	Time ratio between TMT-A-2 and TMT-B-2^e^	0.400		–8.831		–51.693		.002			<.001		<.001		
**Preparation time**															
	TMT-A-1	1.687		3.054		10.412		.01			<.001		<.001		
	TMT-A-2	1.152		3.093		11.485		<.001			.003		.02		
	TMT-B-1	4.174		12.215		15.110		<.001			<.001		.24		
	TMT-B-2	2.046		5.967		6.109		<.001			<.001		.94		
**Execution time**															
	TMT-A-1	11.448		16.375		21.908		<.001			<.001		<.001		
	TMT-A-2	52.498		69.903		78.186		<.001			<.001		.21		
	TMT-B-1	23.174		35.776		24.810		<.001			.55		.001		
	TMT-B-2	136.261		158.356		66.306		.004			<.001		<.001		
**Averaged w** **rite pressure**															
	TMT-A-1	1661.022		1363.905		1107.242		<.001			<.001		.001		
	TMT-A-2	1683.553		1410.751		1013.593		<.001			<.001		<.001		
	TMT-B-1	1674.923		1387.999		871.733		<.001			<.001		<.001		
	TMT-B-2	1684.781		1319.617		554.057		<.001			<.001		<.001		
**Minimal w** **rite pressure**															
	TMT-A-1	477.470		370.000		240.571		.01			<.001		.001		
	TMT-A-2	349.840		212.536		133.041		<.001			<.001		<.001		
	TMT-B-1	308.550		191.268		90.367		<.001			<.001		<.001		
	TMT-B-2	295.510		169.691		58.847		<.001			<.001		<.001		
**Maximal w** **rite pressure**															
	TMT-A-1	1697.950		1431.907		1244.765		<.001			<.001		.03		
	TMT-A-2	1698.570		1434.247		1064.500		<.001			<.001		<.001		
	TMT-B-1	1698.660		1433.515		973.459		<.001			<.001		<.001		
	TMT-B-2	1697.770		1339.216		592.827		<.001			<.001		<.001		
**Jerk**															
	TMT-A-1	39.767		44.539		41.251		.001			.57		.25		
	TMT-A-2	72.802		74.591		57.067		.17			<.001		<.001		
	TMT-B-1	54.051		57.315		37.433		<.001			<.001		<.001		
	TMT-B-2	12.067		17.044		8.814		.14			.08		.03		
**Interactive behaviors^f^**															
	Number of errors in TMT-A-2	0.040		0.144		0.265		.04			.01		.24		
	Number of errors in TMT-B-2	0.430		1.072		0.551		<.001			.45		.01		
	Number of pen-up warnings in TMT-A-2	0.680		1.206		2.561		.003			<.001		.01		
	Number of pen-up warnings in TMT-B-2	0.980		1.629		1.541		.01			.09		.81		
	Number of prompts in TMT-A-2	0.060		0.278		1.490		.002			<.001		<.001		
	Number of prompts in TMT-B-2	1.420		2.959		2.745		<.001			.22		.85		

^a^HC: healthy controls.

^b^MCI: mild cognitive impairment.

^c^AD: Alzheimer disease.

^d^TMT: Trail Making Test.

^e^When the denominator equals zero, it was set to –1 to avoid a divide-by-zero error.

^f^When the participant made a mistake, lifted the pen from the paper, or could not find the next target, the examiner indicated the error, warned, or prompted him or her separately. Interactive behaviors were then counted.

We also explored the development of a supervised machine learning model to validate the efficiency of these features. A 5-fold cross-validation was performed to evaluate the performance and applicability of the decision-making model. First, we implemented and compared different candidate algorithms to screen for a robust learning model, including support vector classification, adaptive boosting, random forest, gradient boosting decision tree, and light gradient boosting machine. The hyperparameters of the classifiers were maintained at default values. In the future, a well-trained model will eventually become a tool for ascertaining patients’ impaired cognition in the clinical environment. As shown in Table 3, the random forest model had the highest accuracy and area under the curve among the popular machine learning algorithms. Hence, the random forest model was used as the final predictive model for further analysis.

Then, we characterized the benefit of mixing paper and electronic TMTs via a quantitative comparison of the performance of conventional features and all the abovementioned features. The former is commonly used in the traditional paper-based TMT, which involves completion time, demographic features (sex, age, and educational level), and the number of interactive behaviors (the number of mistakes, pen-up warnings, and prompts). It is worth noting that the number of interactive behaviors was not incorporated into the proposed feature space because the proposed paper-and-electronic TMT was expected to eliminate dependency on caregivers. A performance comparison is presented in Table 4. This result demonstrates that the proposed feature extraction method is highly beneficial for improving diagnostic ability.

To intuitively highlight the contribution of each feature to the prediction of the cognitive impairment level, we used the Shapley additive explanations (SHAP) method [26] (implemented by the Python package *SHAP*, version 0.41.0) to visualize the variable importance. Specifically, we computed the mean absolute SHAP value for each variable as the importance index. As depicted in Table 5, the proposed features play an important role in cognitive impairment screening, particularly time-related features, writing pressure–related features, and VQ without relative weighting. Meanwhile, the TMT-B provided a better measure of cognitive flexibility and produced more discriminative features than the TMT-A, which is consistent with previous studies.

In addition, to verify the stability and robustness of the proposed method, participants were encouraged to complete the follow-up within 1 year of the first test. We trained the random forest classifier using the initial data collection and validated it during the follow-up assessments (HC group: n=38, MCI group: n=32, and AD group: n=22). Notably, features containing identity-related information, such as sex, age, and educational level, were excluded to avoid data leakage. As listed in Table 6, the well-trained classifier achieved high stability and accuracy in distinguishing the AD group from the HC and MCI groups.

**Table 3:** Comparison of prediction performance of multiple algorithms.

Machine learning algorithm	Accuracy				AUC^a^		
	HC^b^ vs MCI^c^	HC vs AD^d^	AD vs MCI	HC vs MCI		HC vs AD	AD vs MCI
Support vector classification	0.762	0.894	0.790	0.761		0.893	0.791
Adaptive boosting	0.629	0.924	0.764	0.629		0.924	0.765
Random forest	0.726	0.929	0.815	0.725		0.929	0.816
Gradient boosting decision tree	0.726	0.899	0.795	0.726		0.898	0.796
Light gradient boosting machine	0.705	0.914	0.785	0.705		0.914	0.786

^a^AUC: area under the curve.

^b^HC: healthy controls.

^c^MCI: mild cognitive impairment.

^d^AD: Alzheimer disease.

**Table 4:** Diagnostic performance of the random forest model based on the conventional features or all features.

Metrics		HC^a^ vs MCI^b^	HC vs AD^c^	MCI vs AD
**Conventional features^d^**				
	Accuracy	0.716	0.914	0.785
	AUC^e^	0.715	0.913	0.785
	Sensitivity	0.680	0.866	0.745
	Specificity	0.750	0.960	0.826
**Proposed features^f^**				
	Accuracy	0.726	0.929	0.815
	AUC	0.725	0.929	0.816
	Sensitivity	0.701	0.908	0.764
	Specificity	0.750	0.950	0.867

^a^HC: healthy controls.

^b^MCI: mild cognitive impairment.

^c^AD: Alzheimer disease.

^d^Completion time, demographic features (sex, age, and educational level), and the number of interactive behaviors (the number of mistakes, pen-up warnings, prompts).

^e^AUC: area under the curve.

^f^Without the number of interactive behaviors.

**Table 5:** Importance of features for diagnosing brain dysfunction based on the random forest classifier. Since Shapley additive explanations (SHAP) satisfies the key properties of additivity, SHAP values of features within the same group can be aggregated to signify the importance of a whole group of features.

		Absolute SHAP value, mean
**Grouping by TMT^a^ parts**		
	Averaged write pressure (B)	0.036
	Execution time (B)	0.033
	Completion time (A)	0.030
	Completion time (B)	0.029
	VQ^b^ without weights (B)	0.028
	Preparation time (B)	0.027
	DTWD^c^ (B)	0.024
	Execution time (A)	0.020
	DTWD (A)	0.019
	Jerk (B)	0.019
**Aggregating all the features within the same group**		
	Completion time	0.059
	Execution time	0.053
	Averaged write pressure	0.048
	DTWD	0.043
	Preparation time	0.042
	VQ without weights	0.040
	Jerk	0.029
	Minimal write pressure	0.026
	VQ with relative weights	0.015
	Maximal write pressure	0.014

^a^TMT: Trail Making Test.

^b^VQ: vector quantization.

^c^DTWD: dynamic time warping distance.

**Table 6:** Performance of well-trained classifier on the data collected by follow-up within 1 year after the first test. The random forest classifier was trained on the data set from the first test.

Metrics	HC^a^ vs MCI^b^	HC vs AD^c^	MCI vs AD	AD vs others
Accuracy	0.656	0.891	0.857	0.929
AUC^d^	0.659	0.914	0.848	0.902
Sensitivity	0.690	1.000	0.800	0.850
Specificity	0.629	0.829	0.897	0.953

^a^HC: healthy controls.

^b^MCI: mild cognitive impairment.

^c^AD: Alzheimer disease.

^d^AUC: area under the curve.

## Discussion

### Principal Findings

By analyzing 389 participants, we developed and validated a novel combination mode of paper and electronic TMTs, which not only maintained the traditional interaction style for participants who were not familiar or comfortable with electronic presentations but also offered both reproducible and abundant information for automated cognitive assessment. Furthermore, we obtained excellent classification accuracies of 0.726 (HC vs MCI), 0.929 (HC vs AD), and 0.815 (MCI vs AD) using the random forest model. In addition, we also validated our model with the participant’s follow-up data, and we obtained accuracies of 0.656 (HC vs MCI), 0.891 (HC vs AD), 0.857 (MCI vs AD), and 0.929 (AD vs others). We also suggested 4 types of features that were associated with cognitive decline. The experimental results demonstrate the effectiveness of the model in enhancing the accuracy of cognitive impairment screening.

Regarding the correlations between the extracted features and cognitive decline, we used ANOVA to investigate the significance of these features. Both conventional demographic characteristics and time-related features were significantly correlated with cognitive impairment. In addition, the digitization of the TMT allowed us to leverage the recorded data from a different perspective, such as writing pressure and jerk. Writing pressure–related features were significantly correlated with cognitive impairment levels, whereas jerk was slightly correlated. This is in line with a similar previous study [27], which showed that handwriting pressure plays an important role in cognition decline.

The TMT is a widely used neuropsychological test to assess the cognitive function of patients. Sakai et al [28] found that the degree of collapse in the velocity profile shape increased significantly when cognitive function decreased. However, the TMT has limitations: the underlying executive functions articulated during the task are not well discriminated, making it a test with low specificity [29]. Second, in the traditional TMT, only total time is quantified, which does not allow for a detailed analysis. Third, there was a fixed spatial configuration for each condition. We combined the electronic and paper versions of the TMT to overcome these main limitations and evaluated them in a group of older adults with cognitive impairment.

The significance test suggests that the captured features can help improve the performance of automatic screening models, which was also validated by comparing the performance of the machine learning model trained with conventional features and the suggested features. Moreover, the significant differences between the groups indicated that TMT Part B was a more useful tool for measuring the level of cognitive impairment, which is consistent with previous research. A longitudinal study suggested that conversion appears to be less driven by changes in the neurobiological trajectory of the disease than by a sharp decline in functional ability and, to a lesser extent, by declines in executive function [30]. A greater decline in executive function has been shown to be associated with greater ventricular enlargement and volume loss in the frontal, parietal, and temporal lobes [31].

With the development of artificial intelligence and computing technology, the use of digital technology to automatically analyze and assess cognitive function has attracted the attention of researchers because of its objectivity and potential to alleviate the shortage of well-trained physical therapists [5,13,32-34]. Different machine learning algorithms have been used in clinical disease diagnosis, such as deep neural networks [35], logistic regressions, k-nearest neighbors, support vector machines, and naive Bayes classifiers [36]. This study also used 5 common machine learning methods to develop the model. AD is mainly characterized by a dynamic process of neurocognitive changes from normal cognition to MCI and progression to dementia, with the jerk of the TMT also being dynamic. Therefore, in our study, we used a novel trajectory modeling approach based on metric learning (generalized metric learning VQ) [21,37] to extract trajectory features that closely resemble realistic clinical data. This represents a pivotal aspect of our study. The importance of the features also shows that template-based features play an important role in cognitive impairment screening, especially for VQ with relative weighting (one of the top-3 most important features). By incorporating all the features, we observed an Improvement in classification accuracy, suggesting that the electronic TMT features provide more scientifically informative data and hold greater potential for clinical application. We present preliminary evidence suggesting that the proposed combination mode of paper and electronic TMTs is user-friendly, practical, and effective. Our future study plan will focus on the development of realistic applications that hold clinically significant implications for at-home health care.

### Limitations

This study had certain limitations. First, it was a single-center study, and most participants lived in Shanghai. Thus, future research should include multicenter cooperative studies to account for regional and racial differences. Second, our findings were based on the TMT-square and circle, and other types of TMTs remain unexplored. Finally, further research is needed to obtain a clear understanding of how these suggested features relate to the neural changes underlying cognitive impairment.

### Conclusions

We propose a novel combination of paper and electronic TMTs, which is expected to not only retain the traditional interaction style for participants who are not familiar or comfortable with electronic presentations but also offer abundant information for automated cognitive assessment. Further, we have proposed 4 types of features associated with cognitive decline for screening. The results demonstrate the effectiveness of this approach and suggest its potential to contribute to the development of a practical tool for assessing cognitive problems in a clinical environment.

## Abbreviations

AD: Alzheimer disease
DTWD: dynamic time warping distance
HC: healthy controls
MCI: mild cognitive impairment
SHAP: Shapley additive explanations
TMT: Trail Making Test
VQ: vector quantization

## Data Availability

The data sets generated and analyzed during this study are not publicly available due to privacy restrictions but are available from the corresponding author upon reasonable request.
